# White sustainable luminescent determination of nifuroxazide using nitrogen–sulphur co-doped carbon quantum dots nanosensor in bulk and various pharmaceutical matrices†

**DOI:** 10.1039/d3ra05471c

**Published:** 2023-10-11

**Authors:** Mai M. Elnaggar, Amira F. El-Yazbi, Tarek S. Belal, Hadil M. Elbardisy

**Affiliations:** a Department of Pharmaceutical Analytical Chemistry, Faculty of Pharmacy, Alexandria University Alexandria 21521 Egypt amira.elyazbi@alexu.edu.eg +20 34873273 +20 34871317; b Pharmaceutical Analysis Department, Faculty of Pharmacy, Damanhour University Damanhour 22511 Egypt

## Abstract

Nifuroxazide (NFX) is an antimicrobial agent that is frequently used as an intestinal antiseptic and recently was proven to have anticancer properties. This work employs the use of nitrogen and sulphur co-doped carbon quantum dots (NSC-dots) luminescent nanoparticles to propose a highly sensitive, sustainable, white and green spectrofluorometric method for NFX detection in bulk and pharmaceutical dosage forms. l-Cysteine and citric acid were the precursors to synthesize water soluble NSC-dots by a quick and environmentally-friendly hydrothermal process. NSC-dots' native fluorescence was measured at *λ*_em_ = 416 nm following excitation at 345 nm. Addition of NFX resulted in quantitative quenching of NSC-dots' luminescence, which represents the principle over which this luminescent method was based. Additionally, the mechanism of fluorescence quenching was studied and discussed. The analytical procedure was validated according to the ICH-guidelines. Linear response for NFX was obtained in the dynamic range 0.04–15 μg mL^−1^. The estimated NFX detection and quantification limits were 0.005 and 0.015 μg mL^−1^, respectively. The proposed method was employed for NFX quantification into two commercial pharmaceutical dosage forms. The calculated percentage recoveries (*R*%), percentage relative standard deviations (RSD%), and percentage error (Er%) were satisfactory. Comparison with other reported methods showed that the proposed method is superior in several aspects. Evaluation of the whiteness of the proposed method using the RGB 12 algorithm combined with the most widely used greenness evaluation tools, the Analytical Eco-Scale and AGREE, demonstrated its superiority and sustainability over other previously published spectrofluorimetric methods for the assay of NFX in various dosage forms.

## Abstract 2

Nifuroxazide (NFX) fluorimetric determination *via* quenching nitrogen–sulphur co-doped carbon quantum dots (NSC-dots) luminescence intensity.

## Graphical abstract

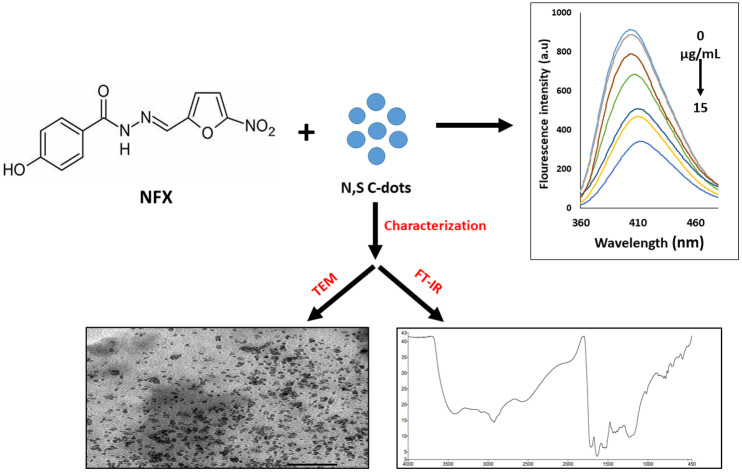

## 1. Introduction

**Fig. 1:**
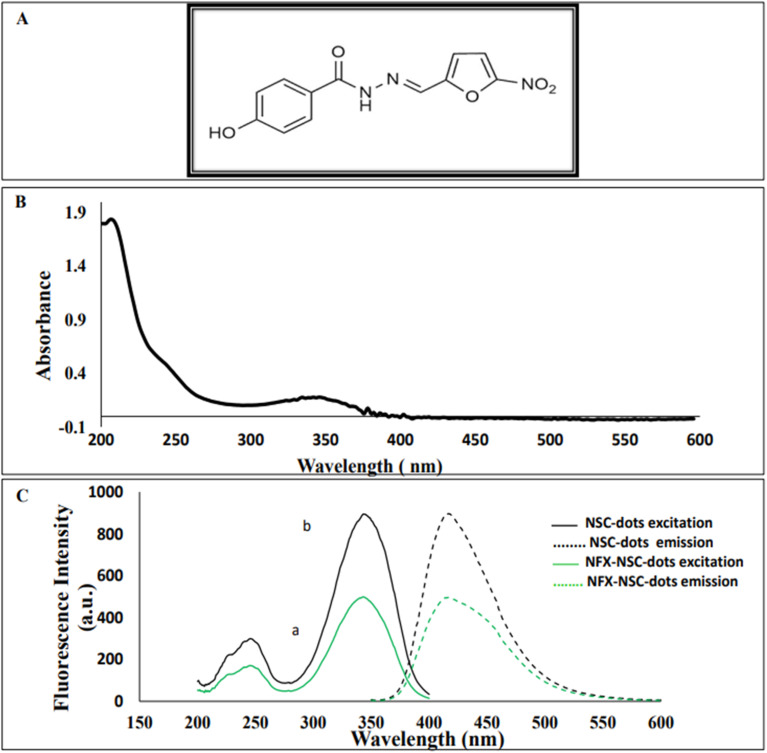
(A) Chemical structure of nifuroxazide (NFX). (B) Absorption spectrum of NSC-dots. (C) Excitation and emission spectra of NSC-dots in the presence (a) and in absence (b) of 5 μg mL^−1^ of NFX (*λ* emission = 416 nm and *λ* excitation = 345 nm).

Nifuroxazide (NFX), 4-hydroxy-*N*′-[(5-nitrofuran-2-yl)methylidene]benzohydrazide (Fig. 1A) is a nitrofuran derivative that possesses broad-spectrum bactericidal action against both Gram-positive and Gram-negative enteropathogenic bacteria. Due to its poor absorption from the digestive system, it is frequently prescribed as an intestinal antiseptic to treat gastroenteritis, acute and chronic diarrhea, and colitis. Moreover, it is used to treat cutaneous and urinary system infections.^1–3^ It is also used for treatment of *H. pylori* infection specially for resistant strains.^4^ Nowadays, NFX has been used as an anticancer medication as it induces cancer cell apoptosis and inhibits tumor growth.^5,6^ It is an official drug in the British Pharmacopoeia^7^ which recommends using 0.1 M NaOH in potentiometric titration for its determination.

By reviewing the literature, it was observed that many analytical techniques have been employed for NFX quantification, such as, spectrophotometry,^8,9^ near IR spectroscopy,^10^ capillary thin layer chromatography,^11^ high performance liquid chromatography,^8,12^ in addition to electrochemical assays.^9,13^ Optically, NFX has an absorption peak at 367 nm, however, it does not possess any native fluorescence. To the best of our knowledge, only three spectrofluorometric studies have been published for the quantification of NFX. The first method was based on formation of a highly fluorescent coumarin compound through the reaction between NFX and ethylacetoacetate using sulfuric acid as a catalyst.^3^ The second method depends on alkaline hydrolysis of NFX by heating with 0.1 M sodium hydroxide solution.^14^ Finally, the third reported assay relied on reduction of NFX with zinc powder in acidic medium which produced a highly fluorescent product.^15^

Special luminous nanosensors known as carbon quantum dots have extraordinary optical and electrical properties along with tiny size and large surface area, making them more promising for use in biomedical and biotechnological applications.^16^ Carbon dots have been widely used as alternative luminescent nanoprobes for quantitative determination of analytes previously assayed by fluorescent dyes or semiconductor quantum dots due to their excellent photostability, tunable emission wavelength, good water-solubility, and low elemental toxicity.^17,18^ Regarding synthesis of luminescent carbon quantum dots; many procedures and methodologies have been developed for this aim, these include: carbonization,^19,20^ chemical and electrochemical oxidation,^21–24^ hydrothermal cutting methodologies,^25^ microwave-assisted methodology,^26^ and others. Whereas, the majority of the previous mentioned methods do not provide a large yield and need expensive equipment hence they are not chosen.

N and S co-doped carbon dots have been widely used in many research groups, as nitrogen has an atomic radius close to that of the carbon atom and has five valence electrons for bonding with carbon atoms in the carbon dots, so it increases the reactive sites of carbon dots.^27^ Also, sulfur atoms can provide energy that modifies the electronic structure of carbon dots and further avoid self-quenching due to their large stokes shift.^27^ The N, S co-doping was found to exhibit excellent fluorescence properties, high quantum yield, high sensitivity, low toxicity and high chemical stability.^27^ N and S co-doped carbon dots have different applications such as the sensitive determination of desperately fluorescent antibacterial drug nitazoxanide and its metabolite in various matrices.^28^ Also, the use of microwave-assisted prepared nitrogen-doped carbon quantum dots was reported for cellular imaging and detection of palbociclib in living cancer cells.^29^ Facile synthesis of biocompatible N, S-doped carbon dots was also published for cell imaging and ion detecting.^30^ In addition, N, S-doped carbon dots were used as sensitive probe for hemoglobin determination.^26^ Moreover, stable nitrogen and sulfur co-doped carbon dots were used for selective folate sensing, *in vivo* imaging and drug delivery.^31^

In this study, a straightforward one-step hydrothermal approach was used to create nitrogen and sulphur co-doped quantum dots (NSC-dots) from l-cysteine and citric acid. Citric acid represented the carbon source, while l-cysteine acted as the source of nitrogen and sulphur.^18^ Herein, the sensitive detection of NFX in bulk powder and in different pharmaceutical dosage forms was accomplished with the help of the synthesized NSC-dots. This suggested luminescent protocol employing the NSC-dots is easy, fast and highly reliable for NFX quantification with sufficient sensitivity. The detection of NFX was based on the quenching effect of the drug on the fluorescence intensity of the prepared NSC-dots *via* inner filter effect and static quenching mechanisms. Furthermore, one of the current key areas of research involves the creation of white and green analytical procedures.^32–39^ For this purpose, the Analytical Eco-Scale procedure,^40^ Analytical GREEnness (AGREE) approach^41^ and white RGB (Red-Green-Blue) 12 model^42^ were employed in this work. Thus, we were able to clarify the sustainability, greenness and the whiteness of the suggested method, specifically, with regard to the consumption of energy, the creation of waste, and dangerous chemicals. By comparing our proposed method with the previously published NFX spectrofluorometric methods, we found that the proposed method is superior, as it is simpler, with high sensitivity, more ecofriendly and it does not require tedious steps. So the designed method can be used as an analytical tool for NFX detection and quantification in quality control labs.

## 2. Experimental

### 2.1. Materials and reagent

NFX (purity% = 99.8 ± 0.82%) was kindly provided by Amoun Pharmaceutical Company, Egypt. The whole study was conducted using analytical-grade reagents. Disodium hydrogen phosphate, boric acid, sodium hydroxide, phosphoric acid, hydrochloric acid, sulphuric acid, dimethyl sulphoxide, methanol, acetone, ethanol, isopropyl alcohol and acetonitrile were purchased from El-Nasr Chemical Company, Cairo, Egypt. Anhydrous citric acid (purity% = 99.9%), and l-cysteine (purity% = 98%) were purchased from Loba Chemie (Mumbai, India). Surface active agents including Tween, cetrimide, and sodium dodecyl sulfate were brought from Sigma-Aldrich, Germany. Commercial pharmaceutical dosage forms, namely, Antinal® oral suspension (labeled to contain 220 mg NFX/5 mL) and Antinal® capsules (labelled to contain 200 mg NFX), (products of Amoun Pharmaceutical Company, Cairo, Egypt), were bought from a local pharmacy. Fresh deionized water was utilized as the diluting solvent throughout the entire work.

### 2.2. Instrumentation and characterization

A Cary Eclipse fluorescence spectrophotometer with 150 W xenon lamp (Agilent Technologies, USA (model: G9800A)) was used to conduct all the spectrofluorometric measurements. A Specord S600 UV-Vis diode array spectrophotometer, associated with 1 cm quartz cells and WinAspect software version 2.3 (Analytik Jena AG, Germany A Cary 360) was used to carry out spectrophotometric measurements. TEM-1400 plus electron microscope was used to examine the synthesized C-dots morphology. Also, Fourier transform infrared (FT-IR) (Agilent Technologies, USA) was employed to record FT-IR spectra. A Thermo Heratherm OGS60 Oven and hydrothermal autoclave reactor, 100 mL stainless steel 316 grade A PTFE were used for NSC-dots synthesis. A Soltec Soluzioni Technology sonicator (Italy, model: 2200EP) and a Crison Instruments SA pH meter (Barcelona, Spain) were utilized during the practical work. All experiments were carried out at ambient temperature.

### 2.3. NSC-dots synthesis

1 g of l-cysteine and 1.82 g anhydrous citric acid were dissolved in a volume of 10 mL deionized water, then a thick syrup was obtained when evaporating it in a hydrothermal autoclave reactor at 70 °C for 12 hours. Afterwards, a heating oven, set at 200 °C, was employed to hydrothermally heat the reaction mixture for three hours at a rate of 10 °C min^−1^. The solution was left to cool at ambient temperature, and the black syrup produced was filtered, then neutralized with 1 M NaOH solution after being diluted with deionized water to 100 mL and sonicated for 5 minutes at ambient temperature before filtration.^18^

### 2.4. Stock solution preparation

NFX stock solution (500 μg mL^−1^) was prepared in 1 mL dimethyl sulfoxide (DMSO) and the volume was completed with acetonitrile. This solution was further diluted using deionized water as a green solvent, in order to generate another two diluted working stock solutions with a concentrations of 50 and 5 μg mL^−1^.

### 2.5. General procedure and construction of the calibration curve

Accurately measured aliquots of NFX standard solution covering the concentration range 0.04–15.0 μg mL^−1^ were transferred into a set of 10 mL volumetric flasks, followed by the addition of 0.1 mL of NSC-dots solution. The volumes were completed to the mark with deionized water and the solutions were mixed well. The fluorescence intensities of the prepared working solutions were measured at *λ*_em_ = 416 nm after excitation at *λ*_ex_ = 345 nm. The readings were subtracted from the corresponding reading of a blank that had received a similar treatment. Each experiment was performed in triplicate. By plotting the difference in luminescence intensity against the corresponding NFX concentrations (μg mL^−1^), the calibration curve was created.

### 2.6. Analysis of pharmaceutical dosage forms

#### 2.6.1. Antinal® oral suspension (220 mg/5 mL)

A 500 μg per mL NFX solution was prepared by pipetting an aliquot of 1.13 mL of Antinal® oral suspension into a 100 mL volumetric flask, drug extraction was fulfilled *via* the addition of 50 mL acetonitrile and 1 mL DMSO to the flask, and following a 15 minute sonication, the solution was diluted to volume with acetonitrile and finally, filtered. This solution was further diluted, in order to generate another two diluted working stock solutions as previously mentioned. Working sample solutions with concentrations within the linearity range were prepared by transferring exact portions of the former filtered solution into a set of 10 mL volumetric flasks and the specified procedure written under “General procedure and construction of the calibration curve” was applied.

#### 2.6.2. Antinal® capsules (200 mg)

The average weight of one capsule was determined by emptying the contents of twenty Antinal® capsules into a weighing boat, homogeneously mixed, and accurately weighed. Following that, the average weight of 1 capsule was added into a 100 mL volumetric flask. In order to extract the drug, 1 mL DMSO and 50 mL acetonitrile were added to the powder, afterwards the solution was left for 15 minute sonication, completed to volume with acetonitrile and ultimately filtered. A step dilution was made by transferring 25 mL from the previous solution into a 100 mL volumetric flask to get 500 μg mL^−1^ as the final drug concentration. This solution was similarly further diluted, in order to generate another two diluted working stock solutions. Different aliquots from the last solution, covering the specified linearity range, were then transferred into a set of 10 mL volumetric flasks and were treated as mentioned under “General procedure and construction of the calibration curve”.

## 3. Results and discussion

### 3.1. Spectral characterization of NSC-dots and method optimization

#### 3.1.1. NSC-dots optical characterization

Herein, a simple, green and economic methodology was applied utilizing NSC-dots for the sensitive analytical quantification of NFX drug. Various spectroscopic and microscopic techniques were used to characterize the prepared NSC-dots. Fluorescence spectroscopy and UV-Vis absorption were used to figure out the optical properties of the synthesized NSC-dots. UV-visible absorption spectra were recorded as illustrated in Fig. 1B. Next, the luminescence spectrum of the prepared NSC-dots was investigated, and as can be seen in Fig. 1C, it has an emission wavelength at *λ*_em_ = 416 nm and an excitation at *λ*_ex_ = 345 nm. On adding 5 μg mL^−1^ of NFX to the NSC-dots solution, its luminescence was extensively quenched as shown in Fig. 1C.

**Fig. 2:**
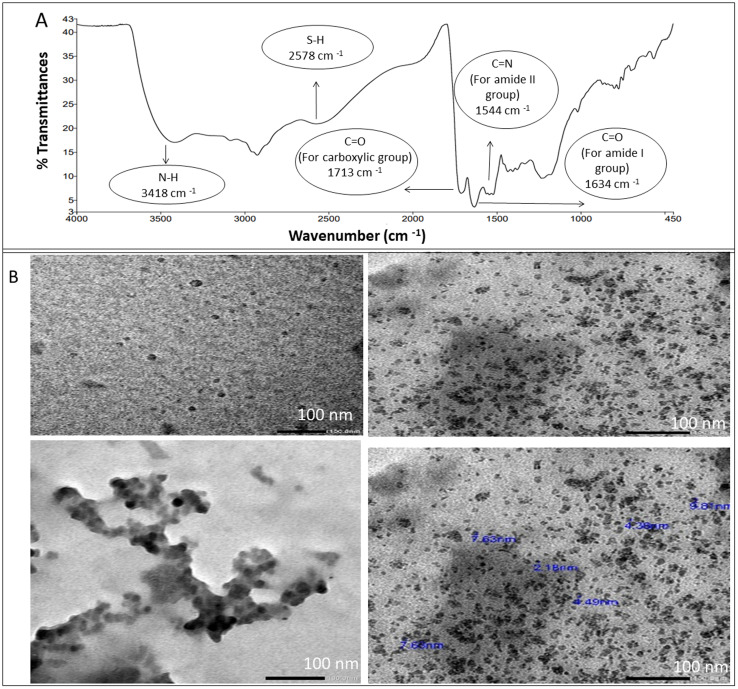
(A) FTIR spectrum of the NSC-dots. (B) TEM images of NSC-dots.

For structural verification of the synthesized NSC-dots, Fourier transform infrared (FTIR) spectra of the NSC-dots was scanned. The FTIR spectra are depicted in Fig. 2A. Spectrum of the NSC-dots showed N–H stretching vibration at 3418 cm^−1^. The carboxylic O–H stretching vibration was accounted for by a very broad absorption band from 3400 cm^−1^ to 2500 cm^−1^. The S–H stretching vibration was observed at 2578 cm^−1^. The C=O stretching vibration for carboxylic acid was observed at 1713 cm^−1^ and for amide carbonyl (amide I) at 1634 cm^−1^. The C=N stretching vibration (amide II band) was assigned to 1544 cm^−1^. To further investigate the morphological characteristics and size of the C-dots, TEM imaging was done. The range of the particle size distribution was 2.18 to 9.8 nm, and Fig. 2B demonstrates the sphere-shaped nature of the synthesized NSC-dots. The characterization data of the NSC-dots obtained herein by UV-visible spectroscopy, fluorescence spectroscopy, TEM and FT-IR corroborate well with the work published by Hadil M. Elbardisy *et al.*^28^

The synthesized C-dots possess a respectably high quantum yield (more than 70%).^18^ This was illustrated by the work published by Dong Y. *et al.*, as the calculated luminescence quantum yield using quinine sulphate as a reference material (activated with 345 nm UV light) was found to be equivalent to 73.0%.^18^ Besides, Dong Y. *et al.*, estimated the quantum yield of the utilized NSC-dots using a second standard, namely 4′,6-diamidino-2-phenylindole (DAPI) dissolved in dimethylsulfoxide, and it was found to be 71.2%.^18^ In addition, the solution remained stable for about 8 weeks without any alteration in its properties.

#### 3.1.2. Investigation of the mechanism of quenching

**Fig. 3:**
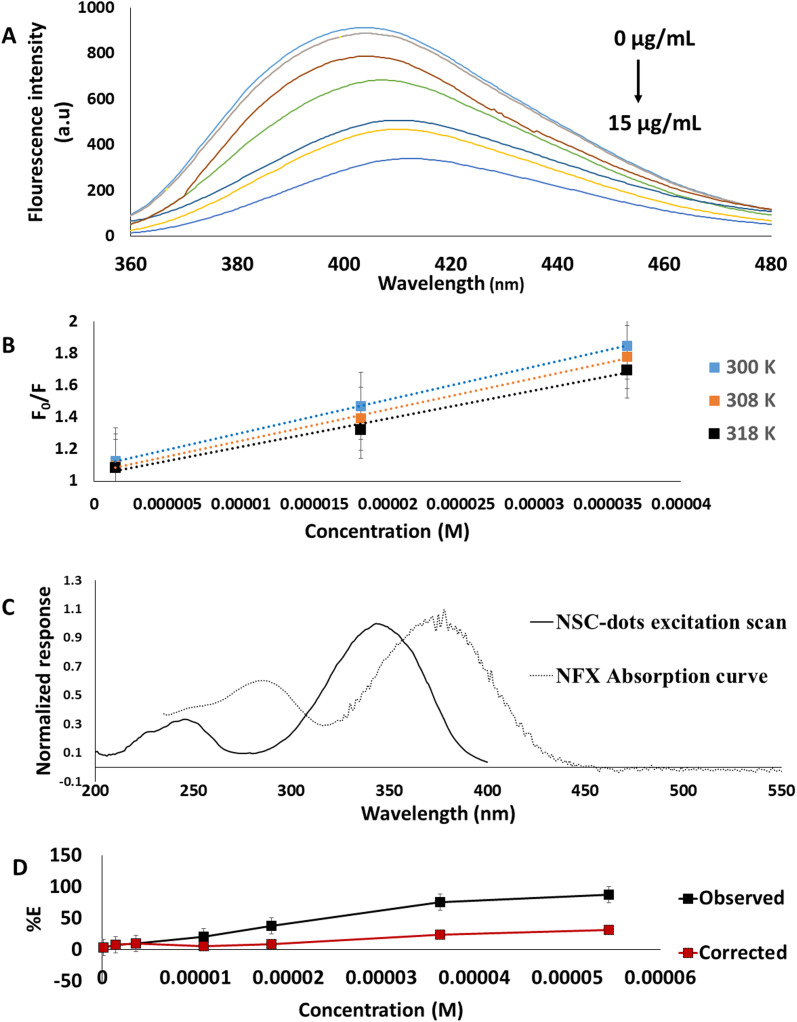
(A) Fluorescence emission spectra of 0.1 mL NSC-dots in aqueous solution upon addition of different concentrations of NFX. (B) Stern–Volmer plot between *F*_0_/*F* and concentration (M) of NFX at different temperatures. (C) Plot showing overlap between UV-Vis absorption spectrum of NFX and fluorescence excitation scan of NSC-dots. (D) Suppressed efficiency (% *E*) of observed and corrected fluorescence of NSC-dots after addition of different concentrations of NFX.

As shown in Fig. 3A, it was discovered that NSC-dots fluorescence quantitatively decreased with increasing concentrations of NFX. The quenching mechanism can generally be divided into a variety of categories, including dynamic quenching, static quenching, and the inner filter effect (IFE).^43,44^

Fluorophore and quencher molecular interaction may cause static or dynamic quenching. The quencher in dynamic quenching diffuses to the fluorophore at the excited state. This quenching does not result in a sustainable change to the molecules. In contrast, a non-fluorescent compound between the quencher and the fluorophore is created during static quenching. It is possible for the quenching to occur through different mechanisms; such as emission group destruction, molecular rearrangements, energy/electron transfer, and others.^45,46^

In order to clarify the potential quenching mechanism, the following Stern–Volmer equation was followed:^28,44,45^*F*_0_/*F* = 1 + *K*_sv_[NFX] = 1 + *K*_q_*τ*_0_[NFX]where *F*_0_ and *F* stands for the fluorescence intensities in the absence and presence of drug, respectively, *K*_q_ represents the bimolecular quenching rate constant, *K*_sv_ refers to the Stern–Volmer quenching constant. The average lifetime (10^−8^ s) is symbolled by *τ*_0_, and [NFX] is the drug molar concentration. The diffusion happens more quickly at higher temperatures, so collisional quenching occurs. On the other hand, static quenching is generally less effective at higher temperatures due to the dissociation of weakly bound complexes.^46^ The static quenching mechanism was proved using the Stern–Volmer plots as the values of *K*_sv_ were calculated at three different temperatures (300, 308, 318 K) Fig. 3B. The values were 2.07 × 10^4^, 1.95 × 10^4^ and 1.75 × 10^4^ L mol^−1^, respectively. Moreover, the values of *K*_q_ for (300, 308, 318 K) temperatures were also calculated, and the results were 2.07 × 10^12^, 1.95 × 10^12^ and 1.75 × 10^12^ L mol^−1^ ·s ^−1^. As these values exceeded the maximum diffusion rate constant (2.0 × 10^10^ L mol^−1^ s ^−1^) by a significant amount,^43^ as a result, it was concluded that, static quenching mechanism was found to be responsible for the NSC-dots fluorescence intensity quenching in presence of the studied drug.^43,44^

For further investigation of the possible quenching mechanism between the synthesized NSC-dots and NFX the inner filter effect was studied. The inner filter effect, previously thought to be an analytical error, has recently begun to establish itself in the field of analysis as a substantial quenching mechanism involving an energy conversion unrelated to radiation.^28,44^ The presence of a complementary inner filter effect was suggested by the overlap between the excitation spectra of NSC-dots and the UV-visible absorption spectrum of NFX Fig. 3B. The inner filter effect was studied using the following equation.^28,43,44^*F*_corrected_ = *F*_observed_ × 10^(*A*^_ex_^+*A*_em_)/2^where; *F*_corrected_ is the corrected fluorescence intensity after removal of IFE, *F*_observed_ is the observed fluorescence intensity, and *A*_ex_ and *A*_em_ are the absorbance of NFX at the excitation and emission wavelengths of the NSC-dots, respectively. The suppressed efficiency (% *E*) for the corrected and observed fluorescence intensity was then determined using the following equation.^28,43,44^% *E* = [1 − (*F*/*F*_0_)] × 100

The results shown in Fig. 3D confirmed that IFE has a role in quenching of NSC-dots fluorescence by NFX since there is a loss of suppression efficiency.

Therefore, it proved that the quenching of the NSC-dots fluorescence intensity in the presence of NFX was caused by both the static quenching mechanism and the inner filter effect.

### 3.2. Method optimization

In order to obtain maximum quenching of the analyzed drug and enhance the assay sensitivity, the reaction's experimental parameters were investigated and optimized. Different factors were studied, including: pH and type of the buffer, buffer volume, effect of surfactant, reaction time and diluent used.

#### 3.2.1. pH and type of the buffer

**Fig. 4:**
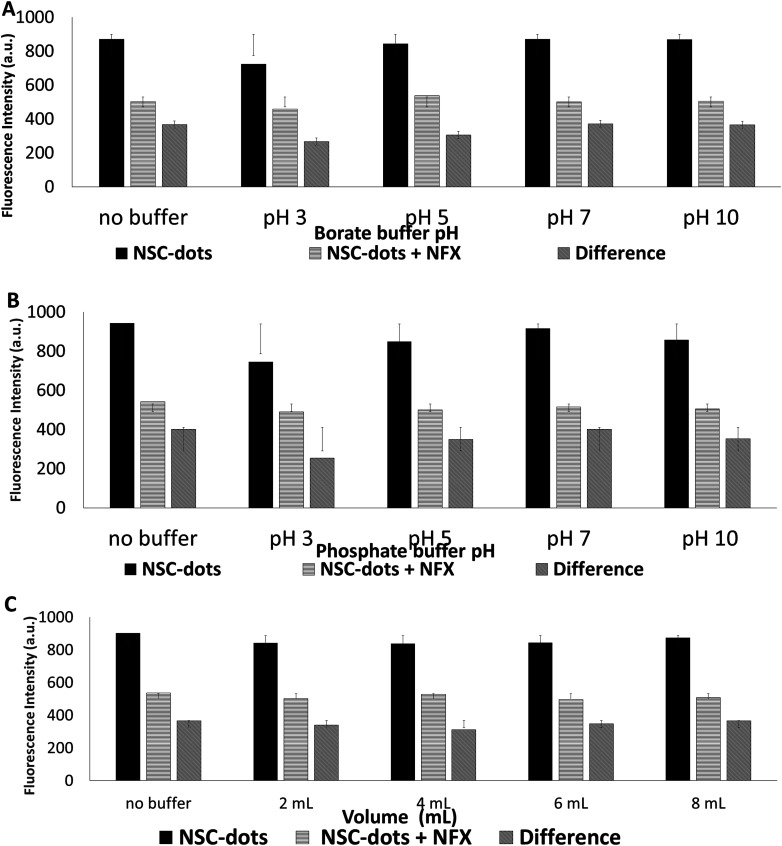
(A) Effect of adding 2 mL of 25 mmol borate buffer with different pHs. (B) Effect of adding 2 mL of 25 mmol phosphate buffer with different pHs. (C) Effect of adding different volumes of 25 mmol borate buffer pH 7.

Two types of buffers, namely borate and phosphate buffer of concentration 25 mM each were studied to examine their effect in improving the method sensitivity. Different volumes from each buffer (2–8 mL in 2 mL increments) and various pHs (pH 3, 5, 7 and 10) were tested and compared to the quenching of NFX–NSC-dots aqueous solution. The results are illustrated in Fig. 4 and S1 (in the ESI†) indicate that no considerable enhancement in NSC-dots quenching was noticed when both buffers were used. This demonstrates that the pH had no considerable effect on the NFX's ability to quench NSC-dots luminescence. Therefore, no buffer was added in this study and thus the proposed luminescent protocol is simpler, whiter, and greener.

#### 3.2.2. Diluting solvent effect

**Fig. 5:**
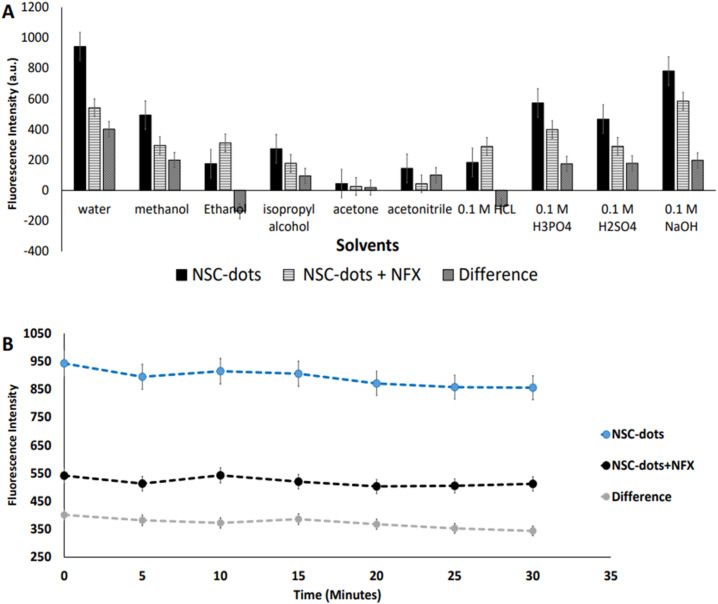
(A) Effect of the diluting solvents. (B) Effect of the reaction time.

The impact of used diluents on the luminescent protocol sensitivity was assessed. This was done by trying a variety of solvents, such as methanol, ethanol, isopropyl alcohol, acetonitrile, acetone, and 0.1 M solutions of each of HCl, H_3_PO_4_, H_2_SO_4_, NaOH and deionized water. Deionized water improved the assay's sensitivity and had the best quenching impact on the NSC-dots, as shown in Fig. 5A and S1.† Basically, this could be explained by the fact that polar solvents lower the π–π* transitions energy and increase the n–π* transition energy, leading to an improvement of the reagent luminescence intensity.^47^ Thus, deionized water was selected as the assay's diluting solvent as it made the procedure more straightforward, more economic and most significantly, made it environmentally friendly.

#### 3.2.3. Reaction time effect

The suppressive effect of NFX on the NSC-dots luminescence intensity was examined as a function of time. The NFX–NSC-dots mixtures were allowed to stand for 30 minutes at room temperature and the luminescence signal was measured every 5 minutes.

As can be seen in Fig. 5B and S1,† when the drug was added to the NSC-dots, an instantaneous reaction occurred immediately (at zero time) and the reaction mixture remained stable to a high extent for 30 minutes. Hence, it was concluded that incubating the drug with the NSC-dots for longer time interval had no effect on quenching NSC-dots luminescence, which speed up and simplified the analytical methodology.

#### 3.2.4. Surfactants effect

**Fig. 6:**
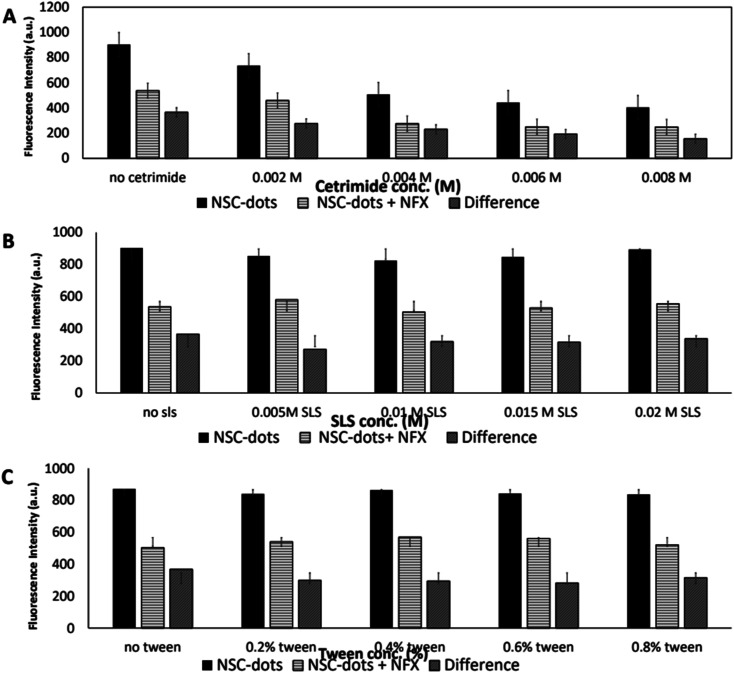
(A) Effect of adding different concentrations of the cationic SAA cetrimide (0.002–0.008 M). (B) Effect of adding different concentrations of the anionic SAA SLS (0.005–0.02 M). (C) Effect of adding different concentrations of the nonionic SAA Tween (0.2–0.8%).

Various surfactants had showed previously that they can affect the fluorescence of C-dots.^48^ Accordingly, various cationic, anionic, and nonionic surfactants were added to the measured solutions to test the effect of NFX quenching. To study the effect of surfactants on the quenching of the suggested luminescent sensor, different types of surface-active agents (*e.g.* cationic, anionic, and nonionic surfactants) were added to the tested solutions and analyzed by the previously mentioned method. We used cetrimide as the cationic surfactant, sodium lauryl sulfate (SLS) as the anionic surfactant and Tween as the nonionic surfactant. Furthermore, various concentrations of the three surfactants were tested. At each concentration, the NFX–NSC-dots' luminescence intensity was measured and compared to that of NFX–NSC-dots aqueous solution. Fig. 6 and S1† results show that the best NFX quenching impact was accomplished without adding any surfactant. Thus, the developed luminescent platform is simpler and greener.

### 3.3. Validation

The International Council for Harmonization (ICH) Q2(R)1 criteria for validation of analytical procedures were followed in order to validate the proposed luminescent protocol.^49^ The studied validation parameters include: concentration range, linearity, detection limit, quantitation limit, accuracy, precision, specificity and selectivity. Tables 1 and 2 summarize all the validation parameters.

**Table 1:** Validation parameters for the spectrofluorometric method suggested for NFX determination

*λ* emission	416 nm
*λ* excitation	345 nm
Linearity range	0.04–15 μg mL^−1^
Intercept (*a*)	37.14
Slope (*b*)	45.19
Standard deviation of the intercept (*S*_a_)	3.16
Standard deviation of the slope (*S*_b_)	0.49
RSD% of the slope (*S*_b_%)	0.01
Correlation coefficient (*r*)	0.9998
Standard deviation of residuals (*S*_*y*/*x*_)	7.54
Variance ratio (*F*)	8359.52
Significance *F*	2.29 × 10^−13^
LOD (μg mL^−1^)	0.005
LOQ (μg mL^−1^)	0.015

**Table 2:** Evaluation of the precision and accuracy for the spectrofluorometric method proposed for NFX determination

Precision & accuracy	Nominal (μg mL^−1^)	Found (μg mL^−1^) ± SD^a^	% RSD^b^	% *E*_r_^c^
Within-day	0.40	0.40 ± 0.005	1.25	0.00
	1.00	1.01 ± 0.01	0.99	1.00
	3.00	2.96 ± 0.01	0.34	−1.33
	10	9.97 ± 0.01	0.10	−0.30
Between-day	0.40	0.40 ± 0.003	0.75	0.00
	1.00	0.99 ± 0.01	1.01	−1.00
	3.00	2.94 ± 0.01	0.34	−2.00
	10.00	10.01 ± 0.01	0.09	0.10

a Mean ± standard deviation for three determinations.

b Relative standard deviation.

c Percentage relative error.

#### 3.3.1. Concentration range and linearity

**Fig. 7:**
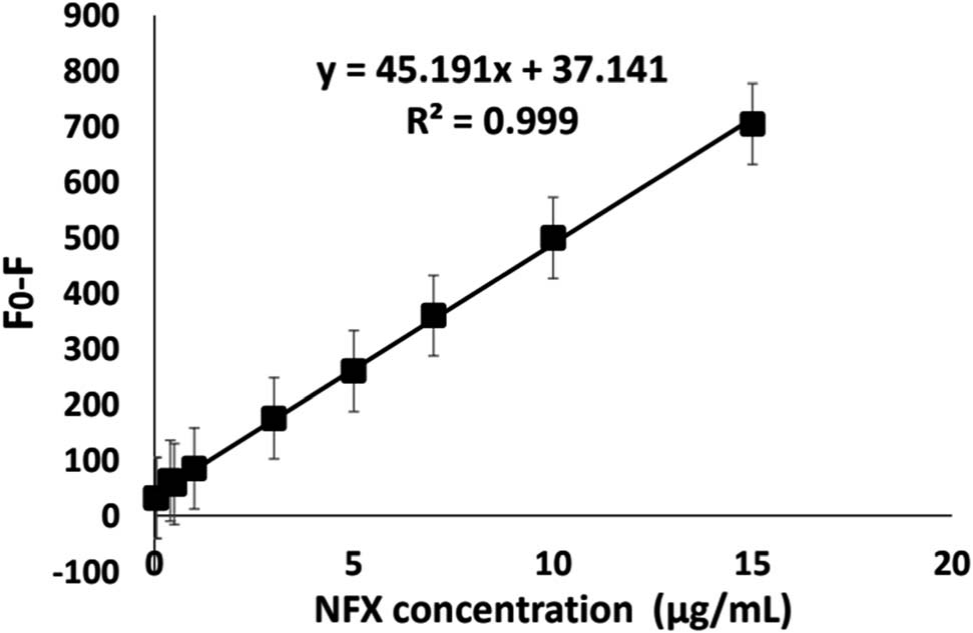
Calibration curve of NSC-dots luminescence quenching against the corresponding NFX's concentrations (μg mL^−1^). *F*_0_: fluorescence intensity of NSC-dots, *F*: fluorescence intensity of NSC-dots + NFX.

After the experimental parameters were studied and optimized, the linearity of the suggested method was evaluated by analysis a series of different NFX concentrations. The calibration curve was constructed by plotting the NSC-dots' luminescence quenching (*F*_0_–*F*; where *F*_0_: NSC-dots luminescence intensity, *F*: NSC-dots + NFX luminescence intensity) against the corresponding NFX's concentrations (Fig. 7), and a linear relationship was obtained in the range of 0.04–15.0 μg mL^−1^. Regression analysis of the results was carried out using the least-squares method for computing values of correlation coefficients (*r*), intercept (*a*), slope (*b*), standard deviation of the intercept (*S*_a_) and slope (*S*_b_). All of the statistical parameters for the suggested procedure are shown in Table 1. The high correlation coefficient value (*r* squared-values > 0.999) together with the small relative standard deviation percentage of the slope (*S*_b_%) (less than 2%) revealed the good accuracy and precision of the linear regression line. Moreover, the low significant *F* value, equivalent to 2.29 × 10^−13^, verifies the limited scatter of the experimental points around the regression line. Also, the low residual standard deviation value (*S*_*y*/*x*_ = 7.54) demonstrates that the plotted points are almost superimposed on the regression line, which supports the good linearity of the suggested method.

#### 3.3.2. Detection and quantification limits

Equations provided by the ICH guidelines were employed in order to calculate the detection limit (LOD) and quantification limit (LOQ). Where LOD = 3.3*S*/*b* and LOQ = 10 *S*/*b*, *S* stands for the standard deviation of six blanks readings (*i.e.*, six C-dots solutions) and *b* represents the slope of the regression line of calibration curve. High sensitivity of the proposed method was confirmed by the low values of LOD and LOQ presented in Table 1.

#### 3.3.3. Accuracy and precision

To test the intra-day and inter-day precision and accuracy of the suggested luminescent protocol, three different NFX concentrations were tested thrice, on the same day and on three consecutive days, respectively. The method's accuracy was confirmed by the acceptable percentage recovery (% *R*) and the low percentage relative error (% *E*_r_) values, which did not surpass 2%. Additionally, the methodology's precision was affirmed by the small percentage relative standard deviation (% RSD) values, which were below 2.0%, illustrating the method's high accuracy and reproducibility for quantifying NFX in bulk powder (Table 2).

#### 3.3.4. Specificity and selectivity

The selectivity of the proposed fluorimetric sensor was evaluated by observing the quenching effect of certain co-administered medications. Such medications include clarithromycin which is indicated for the treatment of *H. pylori* infection^4^ and metronidazole which is used to enhance NFX antibacterial effect.^50^ It was found that both tested medications have no effect on NSC-dots luminescence intensity and thus they did not interfere with NFX fluorimetric analysis. Additionally, the good percentage recoveries presented in Table 3 demonstrate that the excipients and additives co-formulated within the pharmaceutical formulations did not affect the proposed luminescent methodology. Consequently, the method's selectivity and specificity were satisfactory.

**Table 3:** Application of the developed spectrofluorimetric method for determination of NFX in Antinal® oral suspension and capsules

Antinal® capsules (200 mg)			Antinal® oral suspension (220 mg/5 mL)		
Mean% recovery ± SD^a^	RSD%^b^	*E*_r_%^c^	Mean% recovery ± SD^a^	RSD%^b^	*E*_r_%^c^
100.23 ± 1.23	1.23	0.23	99.05 ± 1.41	1.42	−0.95

a Mean ± standard deviation for five % recoveries values.

b Relative standard deviation.

c Percentage relative error.

### 3.4. Pharmaceutical formulation application

The proposed method applicability was assessed by analyzing two NFX commercial pharmaceutical dosage forms present in local pharmacies. The tested pharmaceutical products were: Antinal® capsules (200 mg) and Antinal® oral suspension (220 mg/5 mL) Fig. S1.† External standard approach was adopted for percentage recoveries calculation. The % *R*, standard deviation (SD), RSD%, and % *E*_r_ computed in Table 3 indicate that the proposed methodology is accurate and precise. Furthermore, the co-formulated excipients and additives present in the tested pharmaceutical dosage forms had no effect on the analysis results, demonstrating the high level of specificity and reliability of the suggested protocol.

### 3.5. Evaluation of the method greenness and whiteness

The effects of chemical processes on the environment and health are currently of considerable interest. It was crucial to establish the “greenness” and “whiteness” of the analytical procedures in order to entirely exclude any potential environmental danger. A semi-quantitative approach, named the Analytical Eco-Scale, was employed as a greenness assessment tool.^40^ It depends on estimation of penalty points specified with the Globally Harmonized System (GHS). The total penalty points are subtracted from the best greenness value, which is a hundred “100”. The score of the proposed method was calculated to be 87 (Table 4), while other previously published NFX fluorimetric methods^3,14,15^ have Eco-scale scores in the range 94–75 (Table 4). Thus, the suggested protocol has a satisfactory level of greenness according to the Eco-scale approach. Moreover, a greenness metric software, called “AGREE” assessment tool, offers an objective evaluation of the proposed methodology's environmental effect.^41^ The 12 separate portions of the clock-shaped graph provided by AGREE demonstrate the 12 principles of green analytical chemistry. Each element functions in accordance with a single principle, which is assigned a red, yellow, or green label according on how fully the analytical process is green. The overall assessment value, which ranges from 0 to 1, is shown in the center of the AGREE graph. As shown in Table 4, our proposed luminescent method has an acceptable greenness value within the AGREE pictogram (0.79) in comparison with the other former reported methods (Agree scores in the range 0.80–0.70). Thus, the reported methodology is the greenest among the other reported methods with respect to Agree software.

**Table 4:** Collective analytical comparison between the developed method and other reported spectrofluorimetric methods for NFX analysis

Method	Condition	Linearity range (μg mL^−1^)	LOD (μg mL^−1^)	Greenness and whiteness assessment	
The proposed method	Quenching of the fluorescence of C-dots prepared from citric acid and l-cysteine using deionized water as diluting solvent at (*λ*_ex_ = 345, *λ*_em_ = 416 nm)	0.04–10	0.005	Reagents	Penalty points
				Water	0
				l-Cysteine	1
				Citric acid	1
				Sodium hydroxide	2
				Acetonitrile	4
				Instrumentation	Penalty points
				Spectrofluorometer (<0.1 kW h per sample)	0
				Hydrothermal autoclave reactor (>1.5 kW h per sample)	2
				Occupational hazard (analytical process hermitization)	0
				Waste	3
				Total penalty point	13
				Total score	87
				AGREE metric approach	
				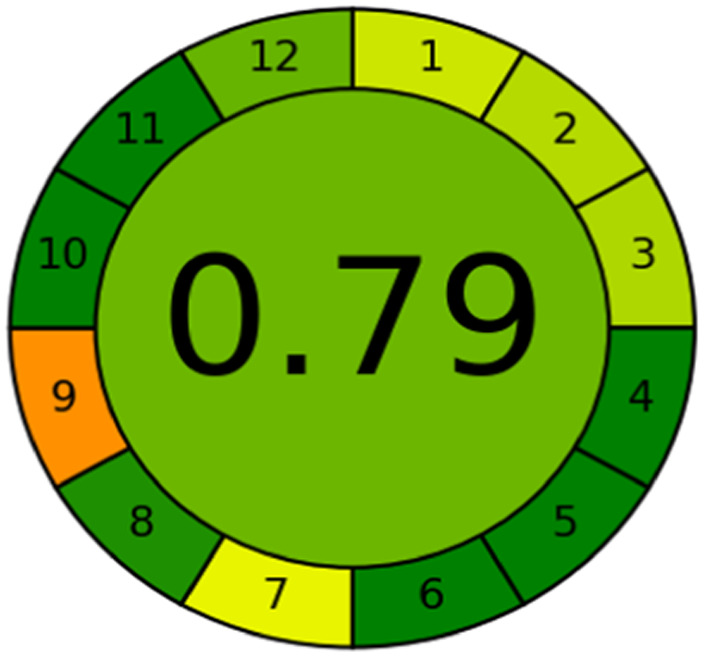	
				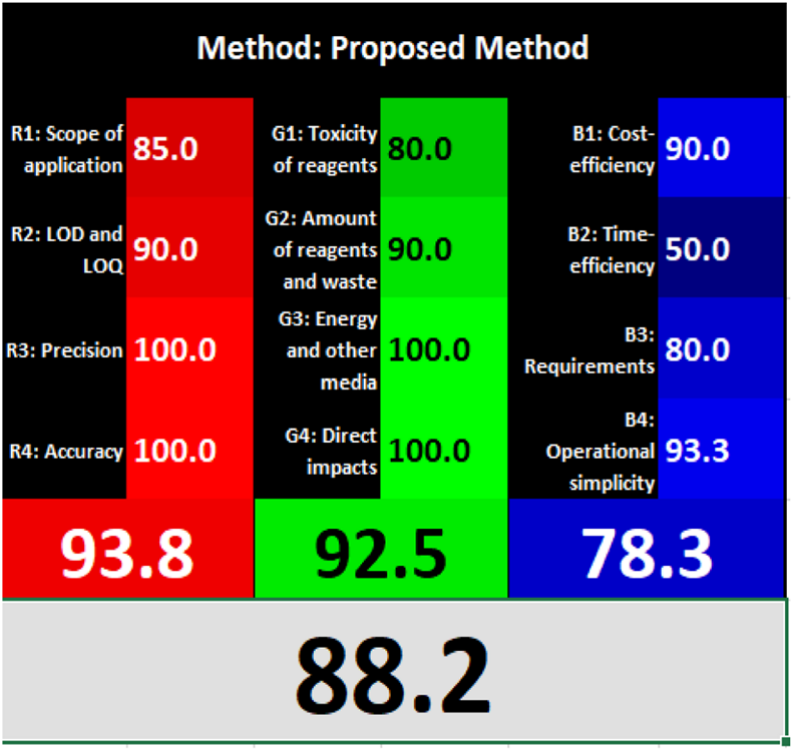	
El-Zaher *et al.*^3^	Formation of a highly fluorescent coumarin compound by the reaction between NFX and ethylacetoacetate using sulfuric acid as catalyst (*λ*_ex_ = 390, *λ*_em_ = 340 nm)	0.02–0.4	0.0001	Reagents	Penalty points
				Ethylacetoacetate	1
				Methanol	12
				H_2_SO_4_ acid	4
				Instrumentation	Penalty points
				Spectrofluorimeter (<0.1 kW h per sample)	0
				Heating in boiling water bath (>1.5 kW h per sample)	2
				Occupational hazard (analytical process hermitization)	0
				Waste	3
				Total penalty points	22
				Analytical Eco-Scale total score	78
				AGREE metric approach	
				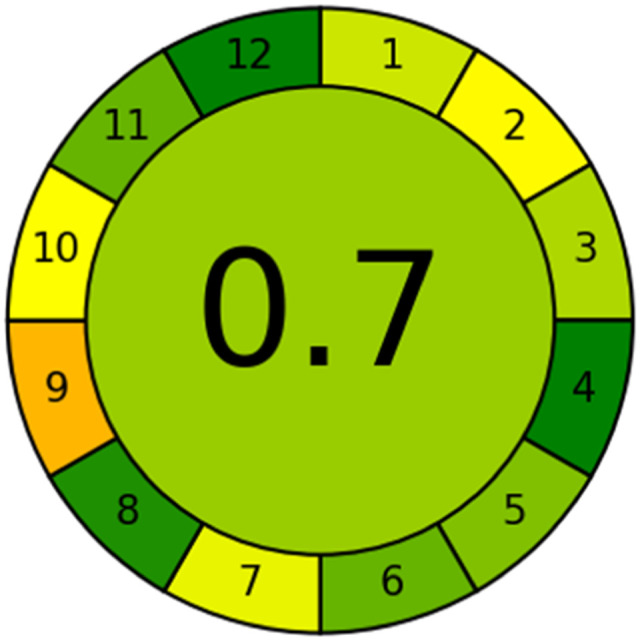	
				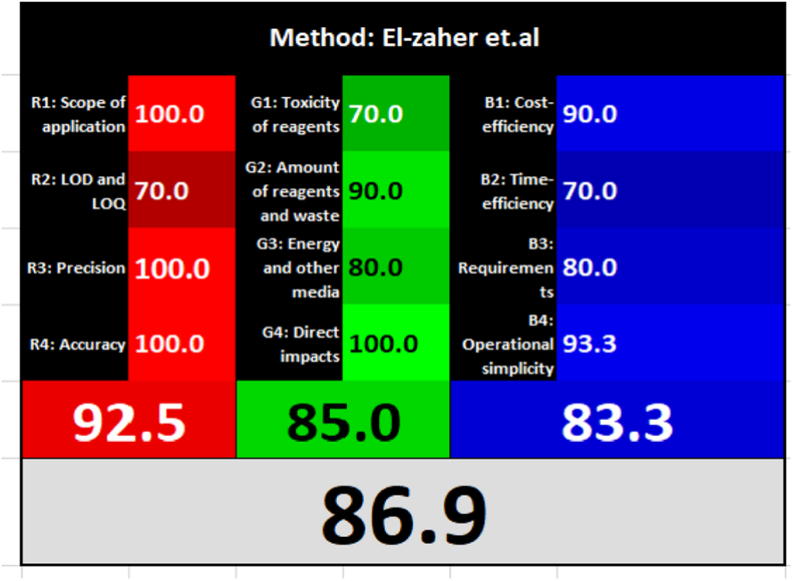	
T. S. Belal^14^	Alkaline hydrolysis of NFX by heating with 0.1 M sodium hydroxide solution (*λ*_ex_ = 265, *λ*_em_ = 465 nm)	0.08–1.00	0.008	Reagents	Penalty points
				Sodium hydroxide	2
				Instrumentation	Penalty points
				Spectrofluorimeter (<0.1 kW h per sample)	0
				Heating in boiling water bath (<1.5 kW h per sample)	1
				Occupational hazard (analytical process hermitization)	0
				Waste	3
				Total penalty point	6
				Analytical Eco-Scale total score	94
				AGREE metric approach	
				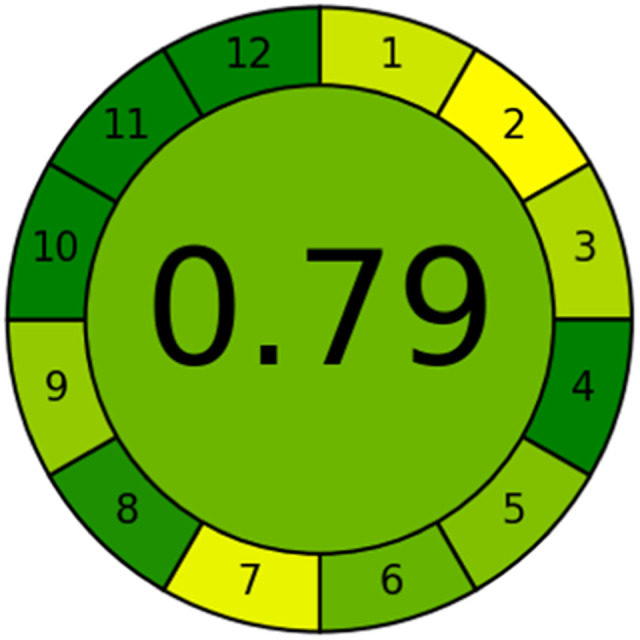	
				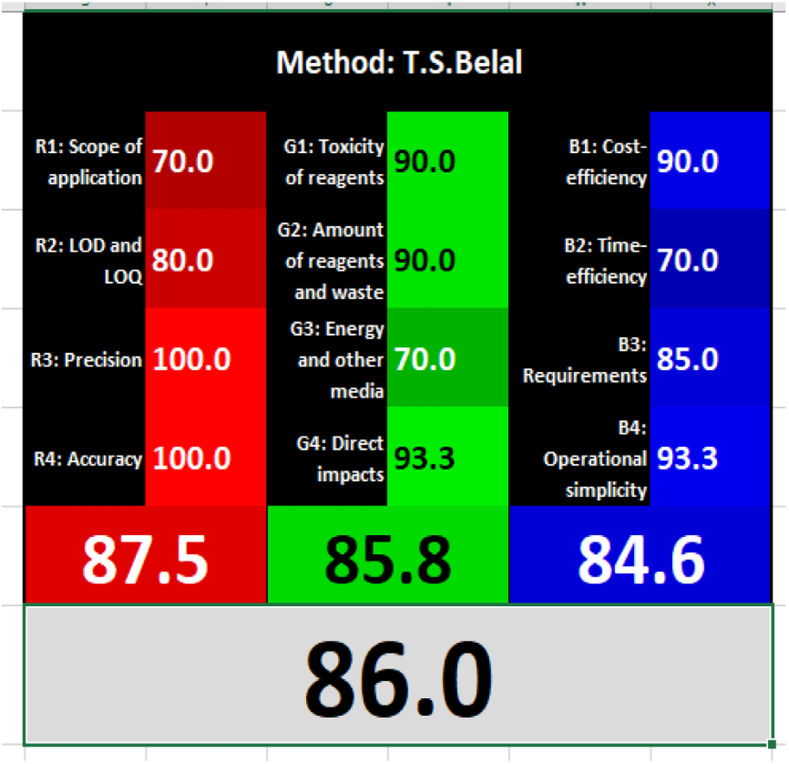	
F. Ibrahim *et al.*^15^	Based on formation of highly fluorescent product upon NFX reduction with zinc powder in acidic medium (*λ*_ex_ = 255, *λ*_em_ = 335 nm)	0.05–1.6	0.0046	Reagents	Penality points
				Zinc powder	2
				Methanol	12
				HCL acid	8
				Instrumentation	Penality points
				Spectrofluorimeter (<0.1 kW h per sample)	0
				Occupational hazard (analytical process hermitization)	0
				Waste	3
				Total penalty points	25
				Analytical Eco-Scale total score	75
				AGREE metric approach	
				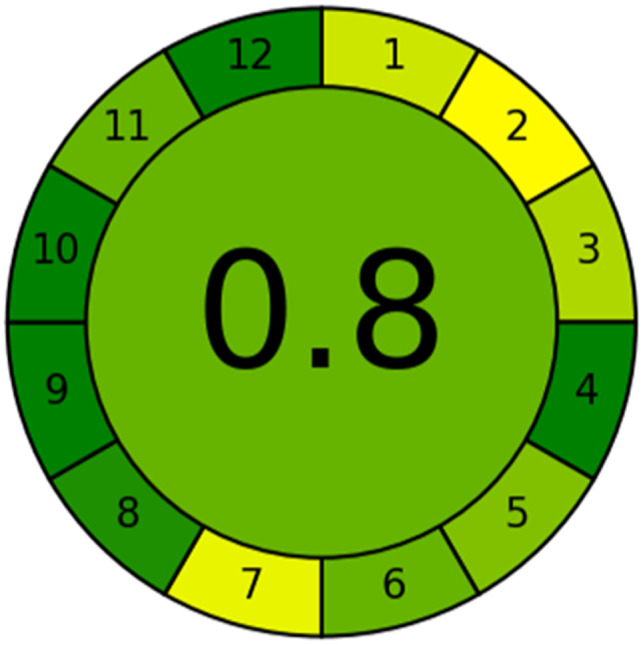	
				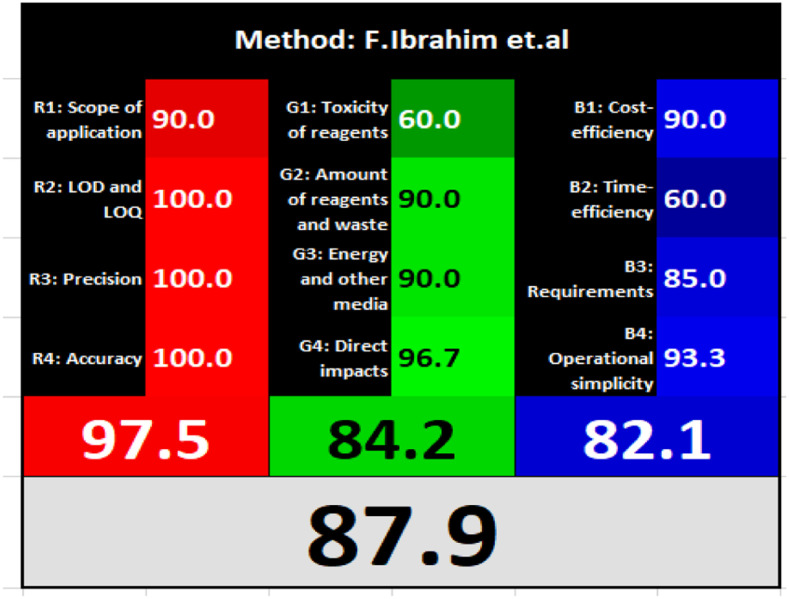	
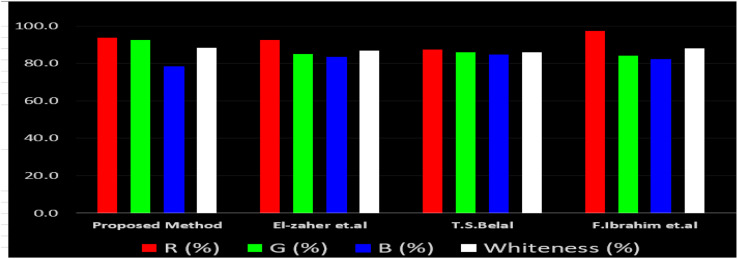					

Likewise, the RGB (Red-Green-Blue) 12 model which is fully integrated with the principles of white analytical chemistry, is presented as Excel spreadsheet.^42^ As an expansion of green analytical chemistry, white analytical chemistry is proposed. As an alternative to the well-known 12 green analytical chemistry principles, 12 white analytical principles were suggested. In addition to green factors, white analytical chemistry considers analytical (red) and practical (blue) characteristics as important factors impacting the quality of the approach.^42^ According to the RGB color paradigm, which states that the appearance of whiteness is created by the combination of red, green, and blue light beams.^42^ RGB 12 score was 88.2% for the proposed method while the other reported methods had scores in the range 86–87.9% (Table 4). Thus, Eco-greenness Scale's assessment, AGREE, and RGB 12 model supported the same results.

### 3.6. Comparison with reported spectrofluorometric methods

El-Zaher *et al.*^3^ published a method for NFX quantification depending on the formation of a coumarin compound which is highly luminescent. This compound is made *via* the reaction between NFX and ethylacetoacetate using sulfuric acid as a catalyst and after heating at 40 °C for 20 minutes in a boiling water bath. In this method methanol was used as diluting solvent.^3^ While, T. S. Belal^14^ method used alkaline hydrolysis of NFX by heating at 60 °C for 20 minutes with 0.1 M sodium hydroxide solution. In this method distilled water was used as a diluting solvent. Additionally, F. Ibrahim *et al*.^15^ reported a method based on the formation of highly luminescent product upon reduction NFX with zinc powder in acidic medium, the reaction mixture was allowed to stand for 30 minutes while shaking, and methanol was used as a diluting solvent.^15^Table 4 demonstrates a comparison between the proposed method with other formerly published fluorimetric methods. The data tabulated in Table 4 show that the suggested method is sufficiently sensitive. In addition, the proposed procedure offers more advantages of being inexpensive, simple and no tedious multi-step procedures are required. Moreover, the suggested method does not require the use of expensive equipment or complex analytical reagents. Additionally, using water as a diluting agent allows the developed method to be a greener and whiter substitute to the other previously reported methods^3,14,15^ present in Table 4. Thus, proving that the designed method is more sustainable and ecofriendly. Also, the proposed method can be a favorable choice for testing NFX purity and routine quality control analysis. Also, the developed method is considered to be non-destructive, as we do not use corrosive acidic or alkaline solutions and we do not need to change pH of the medium. Additionally, the proposed method is time effective where other relevant methods required different time intervals to complete the derivatization reactions.

## 4. Conclusion

In summary, a sensitive, environmentally friendly and cost-effective fluorescent nanosensor has been established for NFX determination. The approach is based on the static quenching of NFX on the fluorescence intensity of NSC-dots and inner filter effect. The preparation procedure of the water soluble NSC-dots is simple, inexpensive and with high yield. It depends on hydrothermal reaction between citric acid aqueous solution with l-cysteine. Compared to the previously reported fluorimetric analytical assays for NFX, this luminescent sensing platform offers a wide range of benefits. Firstly, the lack of costly instrumentation makes this procedure simpler to use and reduces the associated costs. Secondly, this fluorimetric protocol demonstrated good sensitivity as the computed LOD and LOQ values were in the nanoscale level. Advantageously, this enabled NFX quantification in a variety of pharmaceutical dosage forms without interference from usual co-formulated excipients, thus reflecting the method's satisfactory selectivity and reliability. Thirdly, the tailored method employs water as a diluting solvent while using only small amounts of organic solvents. Thus, this work outweighs previously published NFX fluorimetric assays, by virtue of its high greenness and whiteness which represent crucial analytical parameters widely investigated nowadays to ensure the implication of sustainable and green analysis. Accordingly, Analytical Eco-Scale, AGREE metric approach and RGB 12 algorithm were the greenness and whiteness assessment tools adopted in this study to emphasize the eco-friendliness of the suggested fluorimetric nanosensor. Lastly, this method is distinguished by being fast, straightforward and non-destructive as no tedious pre-treatment steps, derivatization reactions or caustic acids or alkalis were included in the procedure, in contrast to previously published methods.

## Data availability

All data will be available upon request.

## Author contributions

M. M. E.: methodology, data analysis, data validation, writing-original draft preparation. A. F. E: supervision, conceptualization, methodology, data analysis, data curation and writing-original draft preparation, reviewing and editing. T. S. B.: supervision, conceptualization, methodology, data curation, investigation and writing-original draft preparation, reviewing and editing. H. M. E.: supervision, conceptualization, methodology, data analysis, data curation and writing-original draft preparation, reviewing and editing.

## Conflicts of interest

The authors declare that there are no conflicts of interest.

## Supplementary Material

RA-013-D3RA05471C-s001
