# Supplementary material for: White sustainable luminescent determination of nifuroxazide using nitrogen–sulphur co-doped carbon quantum dots nanosensor in bulk and various pharmaceutical matrices
Source: RSC Adv. 2023 Oct 11;13(43):29830–46. doi: 10.1039/d3ra05471c (PMC10566585; doi:10.1039/d3ra05471c)
Supplement: RA-013-D3RA05471C-s001 [file RA-013-D3RA05471C-s001.pdf]

Electronic Supplementary Material (ESI) for RSC Advances.
This journal is © The Royal Society of Chemistry 2023

White Sustainable Luminescent Determination of Nifuroxazide using Nitrogen-Sulphur Co-doped Carbon Quantum Dots Nanosensor in Bulk and Various Pharmaceutical Matrices

Supporting Information

Mai M. Elnaggar[a], Amira F. El-Yazbi[a*], Tarek S. Belal[a] and Hadil M. Elbardisy[b]

[a]Department of Pharmaceutical Analytical Chemistry, Faculty of Pharmacy, Alexandria University, Alexandria 21521, Egypt
[b]Pharmaceutical Analysis Department, Faculty of Pharmacy, Damanhour University, Damanhour, 22511, Egypt
[*]Corresponding Author, Tel.: +20 34871317; fax: +20 34873273. E-mail address: amira.elyazbi@alexu.edu.eg

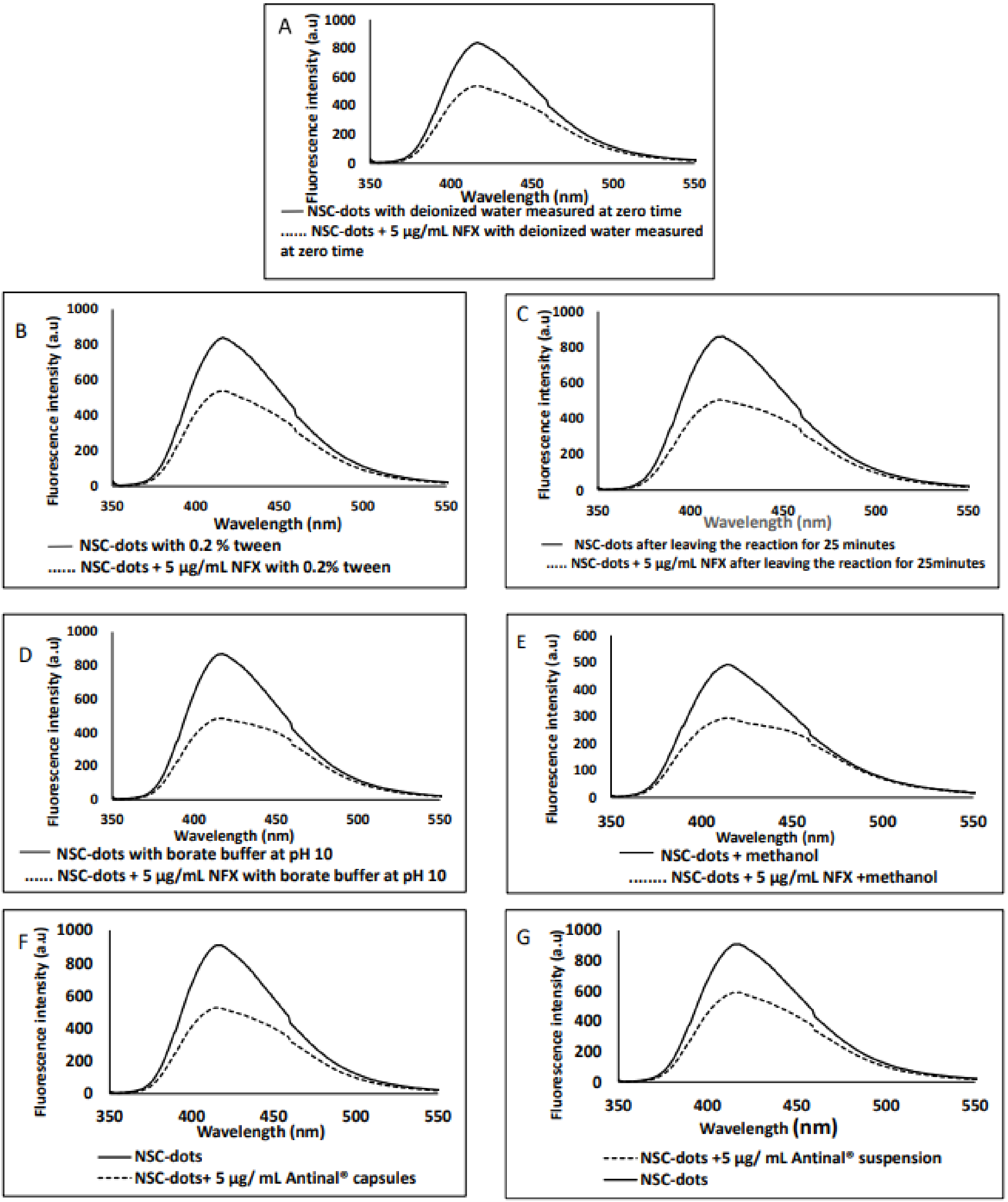

Fig. S1a. Fluorescence emission spectra of NSC-dots and standard 5 µg/mL NFX + NSC-dots in deionized water measured at zero time.

Fig. S1b. Effect of adding 0.2 % of tween on NSC dots and on NSC-dots + 5 µg/mL NFX.

Fig. S1c. Effect of leaving the reaction of NSC dots for 25 minutes and of NSC-dots + 5 µg/mL NFX for 25 minutes.

Fig. S1d. Effect of adding 2 mL of 25 mmolar borate buffer pH 10.
Fig. S1e. Effect of using methanol as diluting solvent for NSC dots and for NSC-dots + 5 µg/mL NFX.
Fig. S1f. Fluorescence emission spectrum of NSC-dots and fluorescence emission spectrum of 5 µg/mL Antinal® capsules+ NSC-dots.
Fig. S1g. Fluorescence emission spectrum of NSC-dots and fluorescence emission spectrum of 5 µg/mL Antinal® suspension + NSC-dots.
